# Effect of propofol on microRNA expression in rat primary embryonic neural stem cells

**DOI:** 10.1186/s12871-016-0259-1

**Published:** 2016-10-13

**Authors:** Jun Fan, Quan Zhou, Zaisheng Qin, Tao Tao

**Affiliations:** Department of Anesthesiology, Nan Fang Hospital, Southern Medical University, Guangzhou, Guangdong China

**Keywords:** Propofol, microRNA, Neural stem cells, Neurogenesis

## Abstract

**Background:**

Propofol is a widely used intravenous anesthetic that is well-known for its protective effect in various human and animal disease models. However, the effects of propofol on neurogenesis, especially on the development of neural stem cells (NSCs), remains unknown. Related microRNAs may act as important regulators in this process.

**Methods:**

Published Gene Expression Omnibus (GEO) DataSets related to propofol were selected and re-analyzed to screen neural development-related genes and predict microRNA (miRNA) expression using bioinformatic methods. Screening of the genes and miRNAs was then validated by qRT-PCR analysis of propofol-treated primary embryonic NSCs.

**Results:**

Four differentially expressed mRNAs were identified in the screen and 19 miRNAs were predicted based on a published GEO DataSet. Two of four mRNAs and four of 19 predicted miRNAs were validated by qRT-PCR analysis of propofol-treated NSCs. Rno-miR-19a (Rno, Rattus Norvegicus) and rno-miR-137, and their target gene *EGR2*, as well as rno-miR-19b-2 and rno-miR-214 and their target gene *ARC* were found to be closely related to neural developmental processes, including proliferation, differentiation, and maturation of NSCs.

**Conclusion:**

Propofol influences miRNA expression; however, further studies are required to elucidate the mechanism underlying the effects of propofol on the four miRNAs and their target genes identified in this study. In particular, the influence of propofol on the entire development process of NSCs remains to be clarified.

**Electronic supplementary material:**

The online version of this article (doi:10.1186/s12871-016-0259-1) contains supplementary material, which is available to authorized users.

## Background

Propofol, a rapid onset intravenous anesthetic, is widely used in general anesthesia induction and maintenance, sedation in intensive care unit (ICU) settings and in various kinds of examinations, such as gastroscopy and pediatric imaging examinations because of its ease of control and comfort recovery attributes. Propofol is also well-known for its neuroprotective effects derived from its anti-oxidant and anti-inflammatory properties. These effects have been demonstrated in a number of different disease models, including post-cardiac arrest brain injury [1] and cerebral ischemia/reperfusion injury [2], in which propofol inhibits the activation of microglia and apoptosis-inducing factor pathway [1, 2] and decreases the production of inflammatory factors [3]. However, there are concerns about the neurotoxicity of propofol. Yu et al. [4] reported that repeated exposure to propofol induced exposure-time-dependent neuronal cell loss and long-term neurocognitive deficits in neonatal rats. Twaroski et al. [5] also demonstrated that propofol induced cell death of human stem cell-derived neurons via a mitochondrial fission/mPTP-mediated pathway. The mechanisms by which propofol produce neuroprotective or neurotoxic effects are still unclear although the effect of propofol on neurogenesis is a focus of research. Based on a rodent cerebral ischemia/reperfusion model, some studies [6, 7] suggested that propofol post-conditioning can promote neurogenesis in the dentate gyrus of the hippocampus, leading to long-term neuroprotection. Interestingly, Engelhard et al. [8, 9] found that propofol may have a minor independent effect on neurogenesis via a cerebral ischemia rat model in 2009, whereas they also demonstrated the toxic effect of propofol on neurogenesis through a traumatic brain injury rat model in 2014. Additionally, Krzisch et al. [10] and Huang et al. [11] provided evidence of the detrimental effects of propofol on adult and early postnatal hippocampus neurogenesis. These controversial results indicating both neuroprotective and neurotoxic effects of propofol may be due not only to the different animal model used by these studies, but also the complexity of the neurogenic process.

MicroRNAs (miRNAs) are small (22–24 nucleotides) non-coding RNAs, which can be incorporated into the RNA-induced silencing complex (RISC) to form the miRNA-loaded RISC (miRISC). Furthermore, the miRISC can bind the 3′ or 5′ untranslated region (UTR) of target mRNAs to induce RNA-based gene silencing. A number of miRNAs have been shown to be related to nervous system development. For example, miR-7 inhibits the NLRP3/caspase-1 axis in adult neural stem cells (NSCs) to promote subventricular zone neurogenesis [12]. MiR-124 and miR-137 affect early neurogenic response through cooperative control of caspase-3 activity [13]. MiR-17/106 targets p38 to modulate neural stem/progenitor cell multipotency [14]. MiR-19 of the miR-17–92 cluster promotes NSC proliferation [15] and targets FoxO1 to regulate NSC differentiation through cooperation with the Notch signaling pathway [16]. MiR-128, miR-132, miR-134, and miR-138 have also been shown to be involved in NSC maturation and dendritic spine morphogenesis [17]. In combination, these data suggest that miRNAs act as not merely as a fine tuning system, but also as key regulators in the development of NSCs during neurogenesis [18]. These findings represent a promising and challenging area of research in the field of anesthesiology. Recently, several investigations showed that miRNAs play pivotal roles in anesthetic-induced neurotoxicity. Twaroski et al. [19] indicated the involvement of miR-21 in propofol-induced cell death via the STAT3/Sprouty-2 pathway using human stem cell-derived neurons. MiR-137, miR-124, miR-34a, and miR-34c have also been implicated in ketamine-induced neurotoxicity in various in vivo and in vitro models [20–23]. Recently, miR-9 was shown to be involved in the inhibition of embryonic stem cell self-renewal and neural differentiation following exposure to the inhaled anesthetic isoflurane [24]. Another investigation also indicated that anxiety-like disorders caused by postnatal exposure to sevoflurane may be related to miR-632, which targets BDNF and a voltage dependent calcium channel [25]. These recent investigations suggest a novel miR-related mechanism responsible for the neurotoxicity of propofol, ketamine, isoflurane and sevoflurane in various in vitro and in vivo models. However, the precise mechanisms are still poorly understood.

In our previous study [26, 27], we found that propofol promotes adult NSC proliferation in vitro but impairs the learning and memory ability of the rats, which may be related to decreased dentate gyrus neurogenesis in the rat hippocampus. Our results, which are consistent with those reported by Krzisch et al. [10], indicated that propofol may have a negative effect on neurogenesis. The development of NSCs in the dentate gyrus, which is a region of neurogenesis in adults, is closely associated with memory and learning ability. Nevertheless, the causes of this apparent contradiction between our previous in vitro and in vivo studies remain to be determined. We hypothesized that miRNAs act as key regulators in these processes; therefore, in this study, we aimed to identify miRNAs that are differentially expressed following exposure to propofol using a non-traditional method based on in-depth analysis of published GEO Datasets. As a result, we confirmed differential expression of four miRNAs in response to propofol treatment.

## Methods

### Microarray datasets and data selection

The Gene Expression Omnibus (GEO) DataSets (http://www.ncbi.nlm.nih.gov/gds) were searched to identify datasets from recent studies (until 06/30/2015) related to propofol anesthesia or sedation in mammalian species and performed using up-to-date whole-genome sequence or microarray chips. We found only one dataset (GEO# GSE4386; Series published: 1/1/2007) [28]. Further data were selected from a total of 10 datasets reported for patients who underwent propofol anesthesia (Table 1). Based on these 10 datasets, the following strategies were used to search the GEO Profiles (http://www.ncbi.nlm.nih.gov/geoprofiles/): 1) propofol and hippocampus; 2) propofol and neural stem cell; 3) propofol and neural stem cell and proliferation; 4) propofol and neural stem cell and differentiation; 5) propofol and neural stem cell and maturation; 6) propofol and neural stem cell and migration; 7) propofol and plasticity; 8) propofol and nerve system development; 9) propofol and brain development; and 10) propofol and learning and memory. The gene list and expression levels were then downloaded from GEO Profiles for further analysis.

**Table 1:** Selected GO biological function and involved genes

GO Term	GO name	*P*-value	Genes
GO:0048167	Regulation of synaptic plasticity	3.20E-06	EGR1, ARC, EGR2, PTGS2, SNCA
GO:0048168	Regulation of neuronal synaptic plasticity	3.37E-05	EGR1, ARC, EGR2, SNCA
GO:0021675	Nerve development	4.84E-04	HOXB3, HES1, EGR2
GO:0007611	Learning or memory	6.42E-04	EGR1, EGR2, PTGS2, TAC1
GO:0030182	Neuron differentiation	0.002551	HES1, EGR2, CXCR4, PHGDH, ID4

### Data screening, bioinformatic analysis and validation

The paired samples of microarray expression data obtained at the beginning and end of bypass surgery from the datasets for the 10 patients were screened according to the following criteria: 1) fold-change in gene expression ≥1.5; 2) fold-change in gene expression ≥1.5 in at least 5 samples; and 3) identical trend of gene expression in the samples. The average fold-change in the expression of the screened genes for each sample were then compiled, and matrix self-organizing map-based clustering analysis of relative gene expression was performed using the R Program.

The screened genes were analyzed by DAVID [29] (the Database for Annotation, Visualization and Integrated Discovery), which is a bioinformatics resource comprising gene and protein annotation databases and several analytical tools for extracting biological relationships from a list of genes. The functional annotation tool of DAVID was used to analyze gene ontology biological function terms enrichment. The genes related in GO terms to neurodevelopment, neural plasticity, learning and memory at *P* < 0.05 were selected for further screening. The frequency of each gene in the selected biological process was determined and genes with frequencies ≥2 were analyzed using the blastn suite of BLAST. The genes with identities ≥85 % between humans and rats were finally selected for qRT-PCR validation.

### MiRNA prediction and screening

MiRWalk2.0 is a comprehensive archive providing a collection of predicted and experimentally verified miR-target interactions with various miRNA databases [30]. The miRNAs which can target the validated genes are predicted using the gene-miRNA interaction information retrieval system of the predicted target module in miRWalk2.0 based on the following databases: miRWalk, miRanda, miRDB and TargetScan. The miRNAs predicted by all four databases were selected for qRT-PCR validation.

### Primary NSC culture and propofol treatment

Time mated pregnant Sprague Dawley rats were anesthetized at gestation day 14 using isoflurane prior to euthanization by cervical dislocation to minimize pain and distress. The embryos were collected, and the cortex and hippocampus of the embryo brains were dissected under a microscope. All animal procedures were approved and conducted in accordance with the guidelines for the care and use of animals of the ethics committee of Southern Medical University. The tissues were the homogenized, digested by Accutase (Millipore, Darmstadt, Germany) and suspended with NSC basal medium (Millipore, Darmstadt, Germany) to form a single cell suspension (2 × 10^6^ cells/ml). The cells were cultured in the NSC basal medium containing 20 ng/ml basic fibroblast growth factor (bFGF; Peprotech, Rocky Hill, USA) and 20 ng/ml epidermal growth factor (EGF; Peprotech, Rocky Hill, USA), and then incubated at 37 °C under 5 % CO_2_ to form neurospheres. At 150–200 μm in diameter, the neurospheres were dispersed into single cells by treatment with Accutase and suspended at the density of 5 × 10^5^ cells/ml. Subsequently, the NSCs were seeded in culture plates or dishes pre-coated with 25 μg/ml poly L-ornithine (Sigma–Aldrich, St. Louis, MO, USA) and cultured for 2–3 days for use in further experiments. The culture medium was then replaced with fresh medium supplemented with 100 mM 2,6-diisopropylphenol (propofol) (Sigma–Aldrich) dissolved in dimethyl sulfoxide (DMSO) (Sigma–Aldrich) at a final concentration of 50 μM. The same procedures were performed using DMSO alone in the control group. The cells were treated for 6 h before being harvested for total RNA extraction at the following time-points: immediately (T1), Day 1 (T2), Day 3 (T3) and Day 7 (T4) after treatment with propofol.

### Immunocytochemistry

The neurospheres and NSCs were identified by immunocytochemistry and the proportion of NSCs was determined by cell counting. The cells were washed once with phosphate-buffered saline (PBS) and fixed for 30 min at room temperature in 4 % paraformaldehyde (Solarbio, Beijing, China) and then 15 min in 0.25 % Triton X-100 (Sigma–Aldrich). After washing three times with PBS, the cells were blocked for 1 h at room temperature in 2 % bovine serum albumin (BSA) (Solarbio) before incubation overnight at 4 °C with anti-nestin (1:300) (Abclonal, Boston, USA) for the detection of NSC as a specific marker of NSCs. The cells were washed three times (10 min each) with PBS and incubated for 1 h at room temperature with FITC-conjugated goat anti-mouse (1:500) (Proteintech, Chicago, IL, USA) secondary antibodies. After the NSCs were washed 3–4 times (5 min each) with PBS, nuclei were counterstained with DAPI (Vectorlabs, Burlingame, CA, USA). Finally, the cells were mounted onto glass slides and imaged using a laser-scanning confocal microscope (Olympus FV10i, Tokyo, Japan) for cell counting using previously described protocols [24]. Briefly, nuclei were counted in five fields per well (center and at the 3, 6, 9, and 12 o’clock positions). Each field contained >100 cells. Nestin-positive and DAPI-positive cells were counted and summed for duplicate wells in three independent experiments. The proportion of NSCs (percentage of NSCs) was calculated as the number of nestin and DAPI double-positive cells divided by the total cells counted ×100 %. Cell numbers were counted by an investigator who was blinded with respect to the sample identity.

### Total RNA extraction and qRT-PCR

Total RNA was isolated from the primary NSCs using TRIzol reagent (ThermoFisher, Waltham, MA, USA) following the manufacturer’s protocol and 1 μg of RNA was used to synthesize cDNA with SuperScriptase III (ThermoFisher) using random primers for mRNA analysis. MiRNAs were isolated using RNAiso for small RNA (TaKaRa, Dalian, China) following the manufacturer’s protocol and 5 μg of RNA was polyadenylated and used to synthesize cDNA with the MirX miRNA First Strand Synthesis kit (Clontech, Nojihigashi, Japan). Expression of mRNA and miRNA was determined by quantitative real-time PCR (qPCR) using the SYBR Green PCR Kit (Qiagen, Duesseldorf, Germany) and MirX miRNA qRT-PCR SYBR Kit (Clontech, Nojihigashi, Japan), respectively. qPCR was performed on the Stratagene Mx3000P Real-Time PCR System (Agilent, Santa Clara, USA) with the following conditions: denaturation at 95 °C for 10 s, followed by 40 cycles of 95 °C for 5 s and 60 °C for 20 s. Three biological samples were each tested in triplicate for each sample. All experiments were repeated three times. GAPDH and U6 were used as endogenous controls for mRNA and miRNA analysis, respectively. The primers sequences used in this analysis are shown in Additional file 1: Table S3. Changes in relative expression were determined using the second derivative maximum method 2^-ΔCT^ calculated by subtracting the cycle threshold (CT) of the endogenous control gene from the CT of the gene of target. Relative fold-changes were calculated using the 2^-ΔΔCT^ method.

### Statistical analysis

Data analysis was performed using SPSS 19.0 software. Data were expressed as mean ± standard deviation (SD). The expression of miRNAs and mRNAs at each time-point was assessed using Student’s *t*-test*. P* values < 0.05 were considered to indicate statistical significance.

## Results

### Candidate genes related to propofol exposure

Based on the search strategies, a total of 420 different genes (Additional file 2: Table S1) that related to propofol exposure were selected when duplicates were excluded and their expression data were downloaded from the GEO Profiles.

**Fig. 1:**
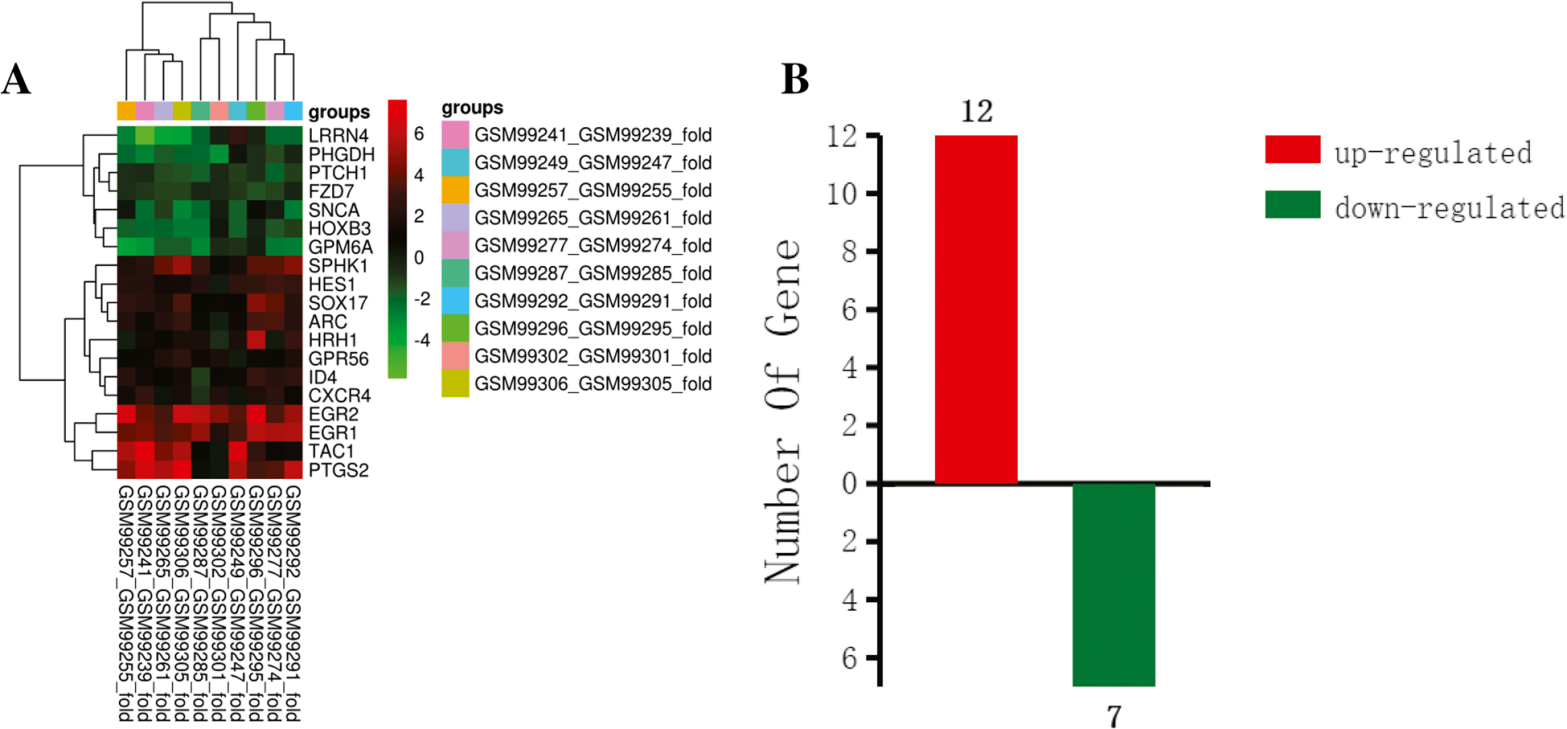
Expression level and number of differentially expressed genes induced by propofol anesthesia. **a** Heatmap of differentially expressed genes induced by propofol anesthesia. *Green* and *red* represent decreased and increased expression, respectively, relative to the average expression in blood samples from patients who received propofol anesthesia. **b** The number of upregulated (*red*) and downregulated (*green*) genes

**Fig. 2:**
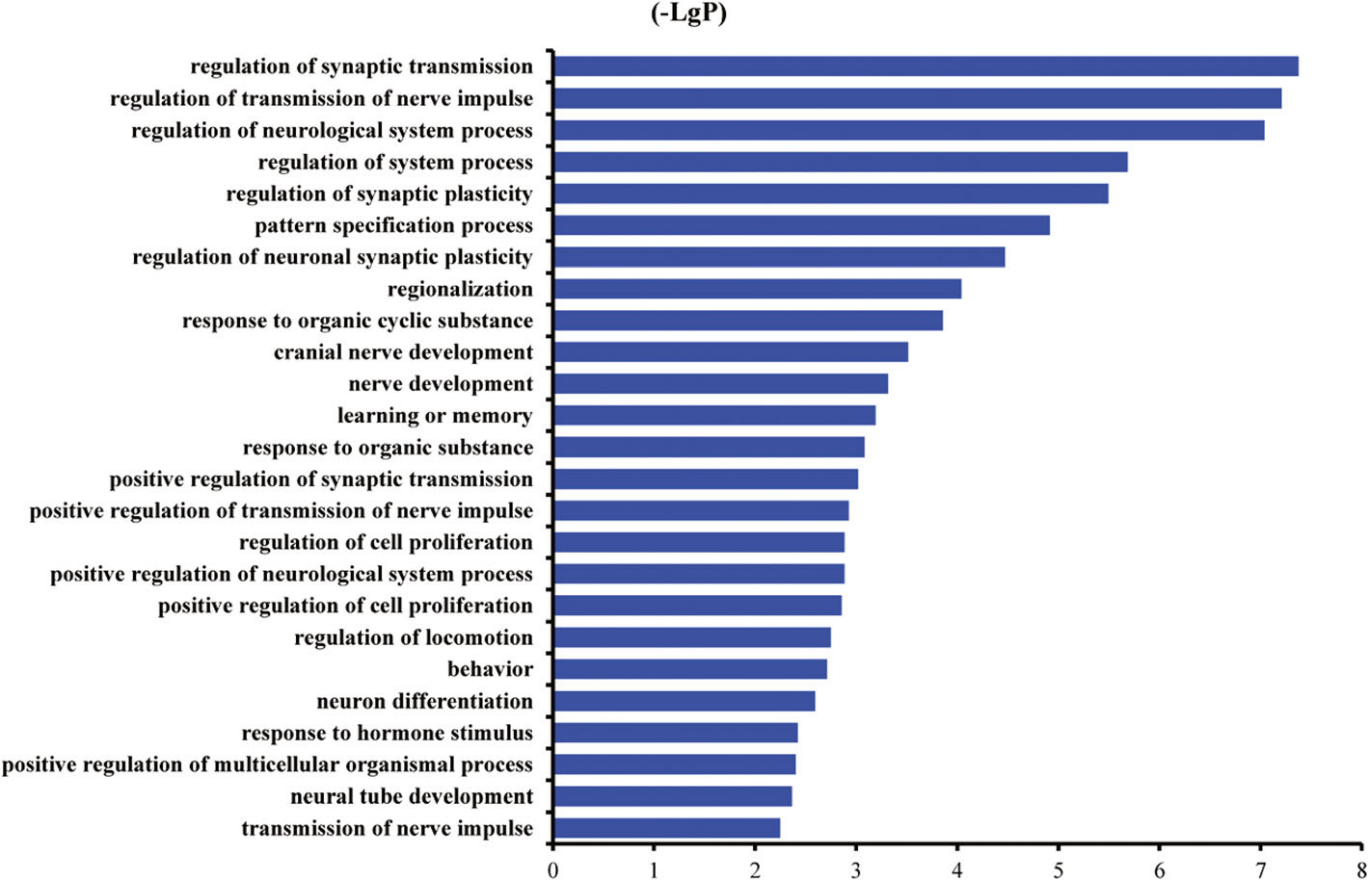
Enrichment of the top 25 biological processes in gene ontology analysis. –LgP is the negative logarithm of the *P*-value, with higher –LgP values indicating greater significance of the biological process

In total, the expression patterns of 19 genes (12 upregulated and 7 downregulated) fulfilled the screening criteria (Fig. 1). We then used DAVID to analyze the gene ontology biological function of the 19 genes. The genes were predicted to be involved in 124 biological functions (Additional file 3: Table S2). Five of these biological functions were related to neurodevelopment, neural plasticity, learning and memory; 11 genes involved in these biological functions were selected for subsequent screening (Table 1). Of these, four genes (EGR1, EGR2, HES1 and ARC) were selected for qRT-PCR validation based on the frequency in the selected biological functions and BLAST identity (Table 2) (Fig. 2).

**Table 2:** The frequency and identity of gene selected for validation

Gene	Count	Expression style	Query	Subject	Identity
EGR2	4	UP	NM_001136177	NM_053633	0.87
EGR1	2	UP	NM_001964	NM_012551	0.86
HES1	2	UP	NM_005524	NM_024360	0.90
ARC	2	UP	NM_015193	XM_008765591	0.86

### Candidate gene expression in propofol-treated NSCs

**Fig. 3:**
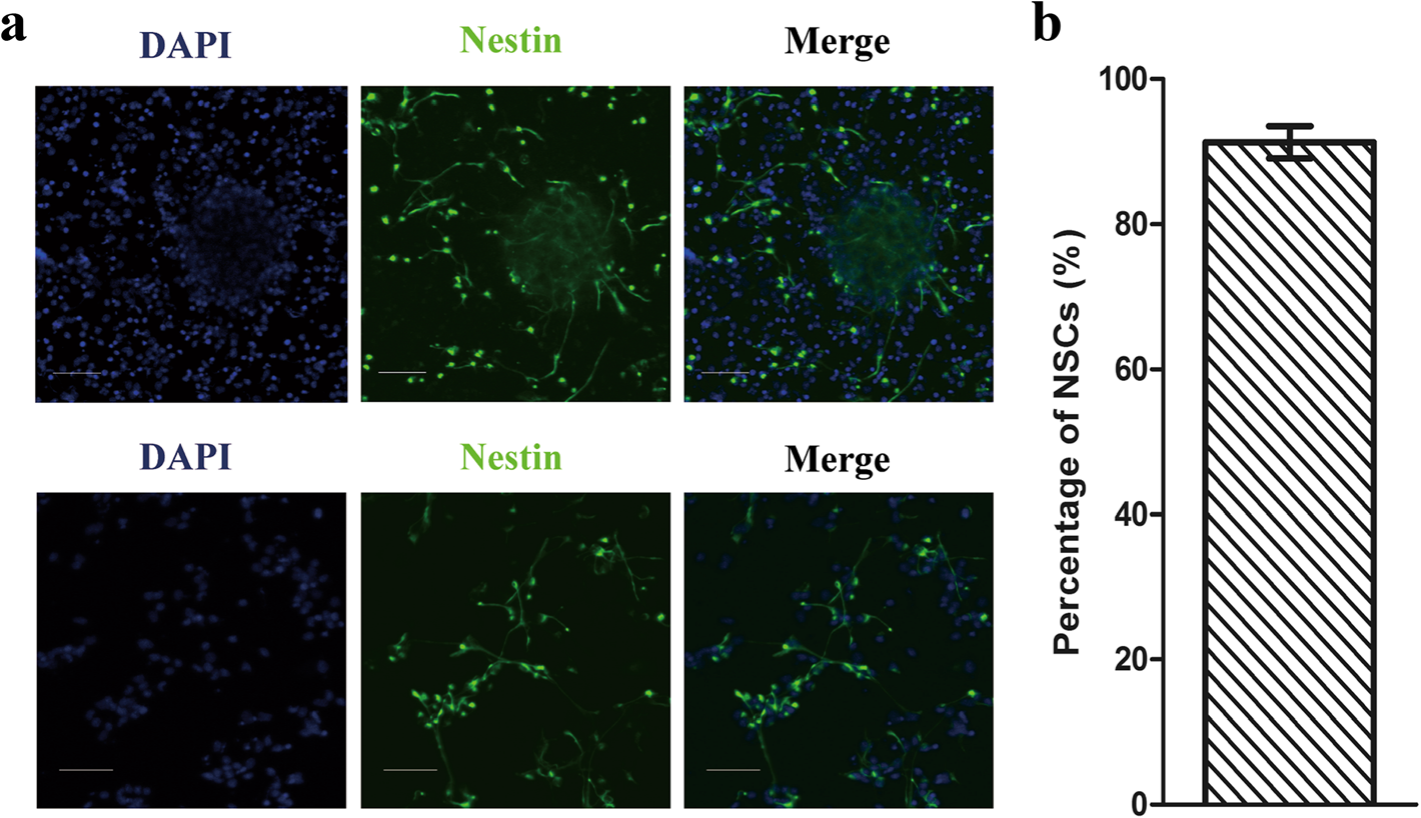
Identification of primary cultured neural spheres and neural stem cells. **a** Neural spheres and neural stem cells were immunostained with anti-nestin antibody (*green*) and counterstained with DAPI (*blue*). **b** Purity of primary cultured neural stem cells calculated by cell counting

**Fig. 4:**
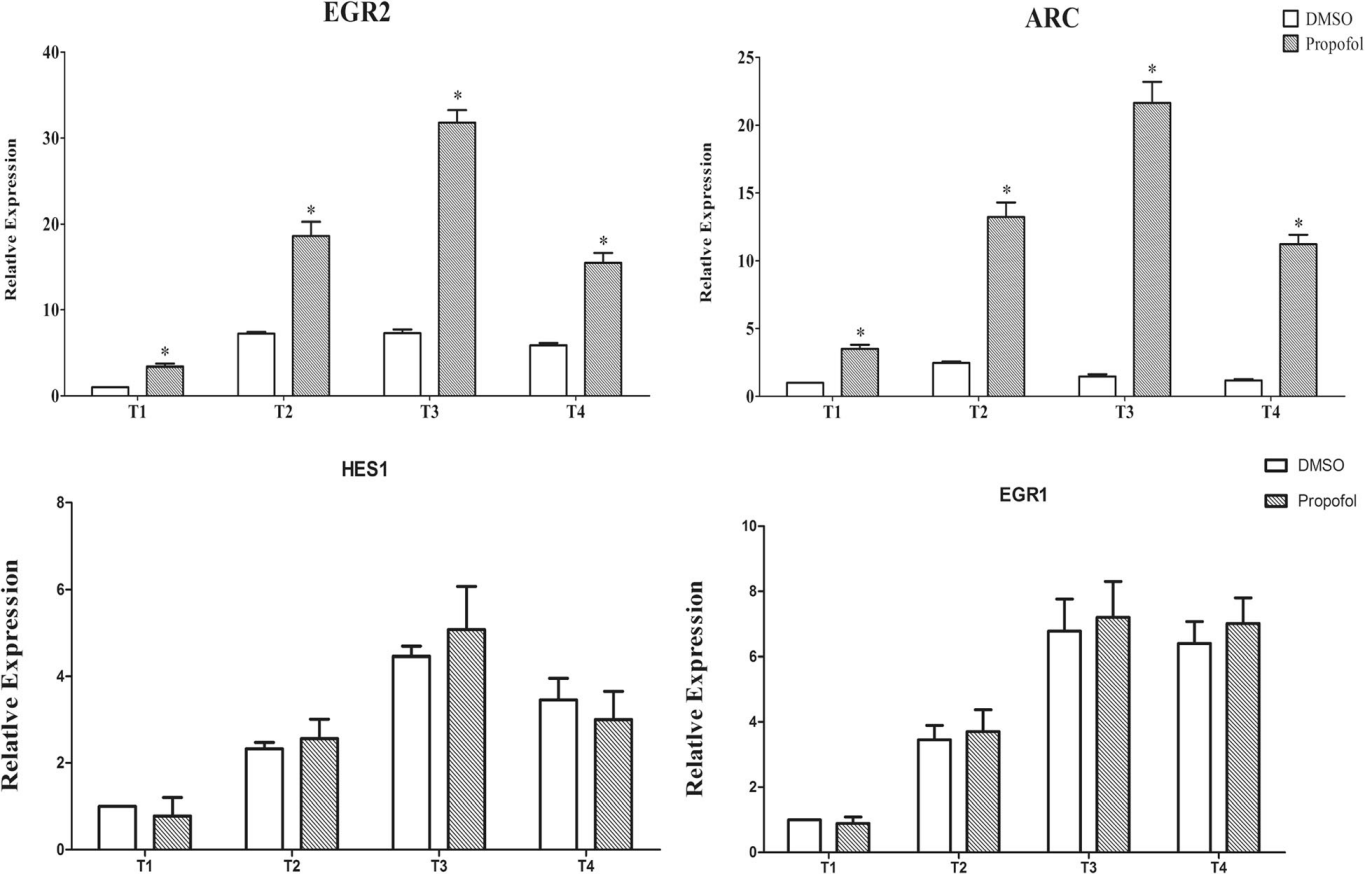
Quantitative RT-PCR analysis of relative EGR2, ARC, HES1 and EGR1 expression. Relative expression levels of EGR2, ARC, HES1 and EGR1 at all four time-points (immediately (T1), Day 1 (T2), Day 3 (T3) and Day 7 (T4) after treatment with propofol or DMSO). *P < 0.05, compared with the DMSO control group at each time-point

Immunocytochemical evaluation showed that the primary cultured neurospheres and NSCs were stained nestin-positive (Fig. 3a). The cell count confirmed that the average proportion of primary cultured NSCs was (91.33 ± 2.24)% (Fig. 3b). Following treatment with propofol for 6 h, significant differential expression of EGR2 and ARC compared with the DMSO control was observed at the four time-points (Fig. 4a and b) whereas there was no significant difference in HES1 and EGR1 expression levels compared with the DMSO control (Fig. 4c and d). Additionally, the fold-change in average relative expression of EGR2 and ARC ranged from 2.58 to 4.38 and 3.48 to 14.76, respectively (Table 3).

**Table 3:** Fold change of EGR2 and ARC expression at four time-points

Gene	T1	T2	T3	T4
EGR2	3.37	2.58	4.38	2.64
ARC	3.48	5.41	14.76	9.57

### MiRNA prediction and expression in propofol-treated NSCs

**Fig. 5:**
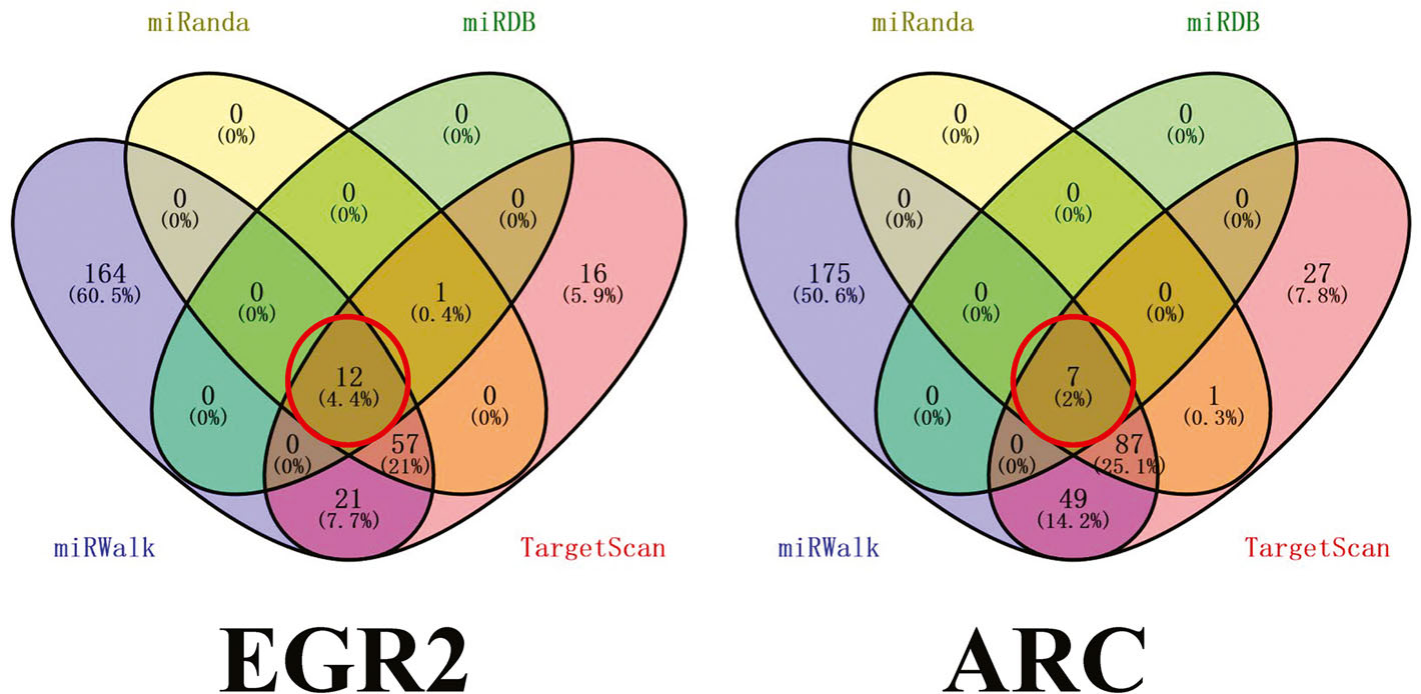
Venn diagram showing the microRNAs predicted to target EGR2 and ARC in the different databases. The digits in the two *red circles* represent the number of microRNAs predicted by all of four databases simultaneously

By searching the four databases (miRWalk, miRanda, miRDB and TargetScan), 248 and 346 miRNAs (Additional file 4: Table S4) were predicted to target the 3′ UTRs of EGR2 and ARC, respectively. The miRNAs predicted by all four databases (rno-miR-19b-2, rno-miR-137, rno-miR-19a and rno-miR-214, (Rno, Rattus Norvegicus)) were selected for validation (Table 4 and Fig. 5).

**Table 4:** Information for microRNAs predicted by four databases to target EGR2 or ARC

Gene	Entrez ID	Refseq ID	MiRNA	MIMATid
EGR2	114090	NM_053633	rno-miR-7a-1-3p	MIMAT0000607
EGR2	114090	NM_053633	rno-miR-3572	MIMAT0017853
EGR2	114090	NM_053633	rno-miR-376b-5p	MIMAT0003195
EGR2	114090	NM_053633	rno-miR-376c-5p	MIMAT0017219
EGR2	114090	NM_053633	rno-miR-145-5p	MIMAT0000851
EGR2	114090	NM_053633	rno-miR-19b-2-5p	MIMAT0017097
EGR2	114090	NM_053633	rno-miR-3591	MIMAT0017893
EGR2	114090	NM_053633	rno-miR-186-3p	MIMAT0017143
EGR2	114090	NM_053633	rno-miR-3065-5p	MIMAT0017839
EGR2	114090	NM_053633	rno-miR-150-5p	MIMAT0000853
EGR2	114090	NM_053633	rno-miR-137-3p	MIMAT0000843
EGR2	114090	NM_053633	rno-miR-224-5p	MIMAT0003119
ARC	54323	NM_019361	rno-miR-19b-3p	MIMAT0000788
ARC	54323	NM_019361	rno-miR-664-2-5p	MIMAT0017229
ARC	54323	NM_019361	rno-miR-219b	MIMAT0017882
ARC	54323	NM_019361	rno-miR-214-3p	MIMAT0000885
ARC	54323	NM_019361	rno-miR-632	MIMAT0012837
ARC	54323	NM_019361	rno-miR-19a-3p	MIMAT0000789
ARC	54323	NM_019361	rno-miR-664-1-5p	MIMAT0017228

**Fig. 6:**
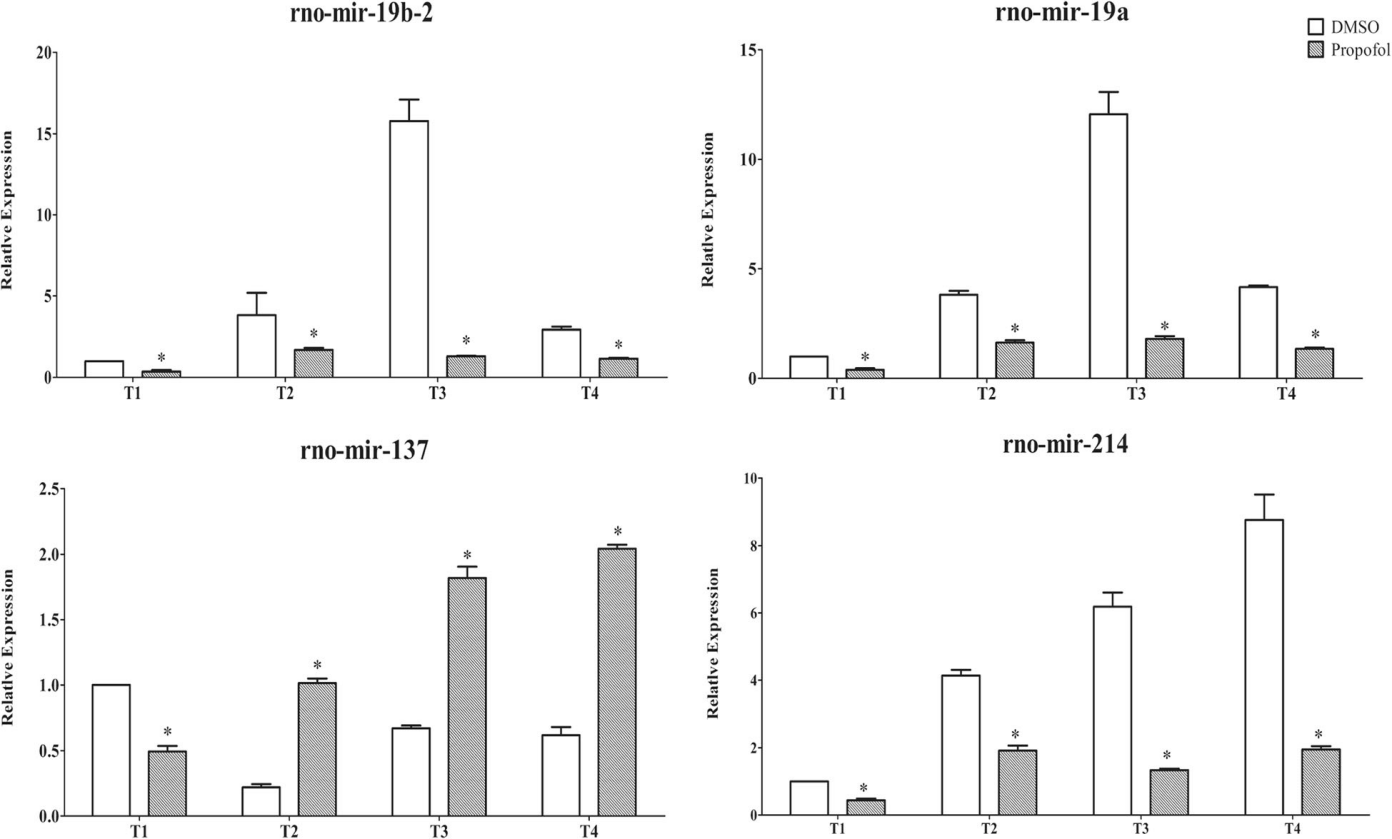
Quantitative RT-PCR analysis of relative expression levels of rno-miR-19b-2, rno-miR-137, rno-miR-19a and rno-miR-214. Relative expression levels of rno-miR-19b-2, rno-miR-137, rno-miR-19a and rno-miR-214 at all four time-points (immediately (T1), Day 1 (T2), Day 3 (T3) and Day 7 (T4) after treatment with propofol or DMSO). *P < 0.05, compared with the DMSO control group at each time-point

Following treatment with propofol for 6 h, significant differential expression of the four selected miRNAs compared with the DMSO control was observed the four time-points (Fig. 6). Rno-miR-19a, rno-miR-19b-2 and rno-miR-214 were downregulated at all four time-points, while rno-miR-137 was downregulated at T1 followed by upregulation from T2 to T4 (Fig. 6). The fold-change in the mean expression levels of rno-miR-19b-2, rno-miR-137, rno-miR-19a and rno-miR-214 ranged from -2.56 to -12.15, -2.02 to 4.61, -2.33 to -6.68 and -2.16 to -4.63, respectively (Table 5).

**Table 5:** Fold change in microRNA expression at four time-points

MicroRNA	T1	T2	T3	T4
rno-miR-19b-2-5p	−2.80	−2.26	−12.15	−2.56
rno-miR-137	−2.02	4.61	2.72	3.32
rno-miR-19a-3p	−2.56	−2.33	−6.68	−3.09
rno-miR-214	−2.30	−2.16	−4.63	−4.50

The minus symbol (−) indicates downregulation

## Discussion

In the current search of PubMed, we found no more than 25 articles directly related to the interaction between propofol and miRNAs. Two of these [31, 32] described the miRNA expression profiles of rat hippocampus and cortex after propofol and sevoflurane anesthesia. In two articles by Pei et al., one described the miRNA expression profiles of developing rat hypocampal astrocytes after propofol treatment [33] and the second reported that propofol upregulates rno-miR-665 expression to induce apoptosis in developing hippocampal astrocytes via a rno-miR-665/BLC2L1/caspase-3-mediated mechanism [34]. Gomez-Martin et al. [35] suggested that propofol induces death in the neurons derived from human stem cells and downregulates miR-21 via a mechanism that is likely to involve STAT3 activation and Akt downregulation. These results provide evidence that propofol treatment causes changes in miRNA expression. However, as of the end of July 2016, there are no reports describing the effect of propofol on miRNA expression in NSCs.

EGR2 (early growth response-2), which is a member of the EGR family, acts as a key regulator in immune tolerance [36]. The function of EGR2 in NSCs is still unknown. Parkinson et al. found that EGR2 (Krox-20) expression exerts a strong protective effect against cells apoptosis and safeguards Schwann cells from death by growth factor deprivation [36]. This result is consistent with those of our previous study showing that propofol treatment reduces apoptosis and promotes proliferation of adult NSCs [26]. In our current study, EGR2 mRNA in NSCs was elevated immediately after treatment with propofol for 6 h and this upregulation persisted to 7 days after treatment. Therefore, we speculate that the elevated EGR2 expression may contribute partly to the proliferation of NSCs observed in vitro in our previous report [26]. On the other hand, the decrease in neurogenesis reported both by Krzisch et al. [10] and ourselves [27] may result partly from EGR2 upregulation, which promotes myelination and induces NSC differentiation into oligodendrocytes in the central nervous system. Of the two miRNAs predicted to target EGR2, miR-19b was downregulated, which in accordance with the increased expression of EGR2, while miR-137 was downregulated only at T1 followed by upregulation from T2 to T4. MiR-19b is a member of miR-19 family located in the miR-106–25 cluster, which has been reported to be involved in regulating NSC proliferation and differentiation through a network related to the insulin/IGF-FoxO pathway [37]. MiR-137 is a versatile miRNA that plays different roles in the proliferation, differentiation and maturation of NSCs. Shi et al. found that miR-137 exerts a negative effect on proliferation of embryonic NSCs and then accelerates differentiation via a feedback regulatory loop with TLX and LSD1 [38]. A previous study also demonstrated that miR-137 promotes proliferation and represses differentiation of NSCs by targeting Ezh2 [39] and regulates NSC maturation by targeting mind bomb-1 [40]. The expression patterns obtained in the present study combined with the results of these previous reports indicate that miR-19b and miR-137 interact with EGR2 to promote proliferation and repress the differentiation of NSCs. However, the potential relationship between EGR2 and miR-19b/miR-137 on the development of NSCs remains to be fully elucidated.

ARC (activity-regulated cytoskeleton-associated protein) is another key factor in early embryonic development. As a member of the immediate-early gene (IEG) family, ARC plays a critical role in learning, memory consolidation [41] and synaptic plasticity [42] and acts as a regulator in cell morphology, cytoskeletal organization and cell migration [43]. The activation of cAMP promotes ARC expression [44, 45]. The upregulation of ARC expression observed in our present study may be due to the propofol induced CREB phosphorylation that we reported previously [26]. Furthermore, we observed downregulated expression of miR-19a and miR-214, which are predicted to target ARC. MiR-19a is located in the miR-17–92 cluster, which promotes the NSC proliferation via repression of PTEN [15]. Another study conducted in a murine stroke model confirmed that miR-19a upregulation promotes NSC proliferation by targeting PTEN [46]. MiR-214 is located in the miR-199a–214 cluster and targets PTEN to produce a protective effect in cardiac myocytes against H_2_O_2_-induced injury [47]. Lee et al. reported that miR-214 may act as a novel intermediator in controlling the NSC development [48]. Recently, Huat et al. [49] found that the miR-214 was downregulated during the development of neural progenitor-like cell derived from rat bone marrow mesenchymal stem cell induced by IGF-1, bFGF and EGF. It can be speculated that the increased expression of ARC in NSCs following exposure to propofol will have a beneficial effect, which is in conflict with the neurotoxic effects of propofol in vivo reported by Krzisch et al. [10] Furthermore, when combined the patterns of miR-19a and miR-214 expression, the situation is much more complex and the results are somewhat contradictory. Clearly more sophisticated studies are required to explain these paradoxical phenomena and the underlying mechanism.

In this study, we did not adopt the traditional approach of miRNA sequencing or miRNA array analysis to screen the differences in miRNA expression of NSCs after propofol treatment. Instead, we developed a method based on re-analysis of a published GEO DataSet from a study which aimed to identify myocardial transcriptional phenotypes after propofol and sevoflurane anesthesia to predict cardiovascular biomarkers and function in patients undergoing off-pump coronary artery bypass graft surgery [28]. In that study, the authors compared and analyzed the different gene expression profiles of blood samples biopsied from two time-points, at the beginning and at end of the surgery. Therefore, we hypothesized that most of these genes are also expressed by brain tissue and designed a set of criteria to screen or predict the NSC-related genes or miRNAs. In our study, four candidate mRNAs and 19 candidate miRNAs were selected for qRT-PCR validation. In this way, we confirmed two genes (EGR2 and ARC) and four miRNAs (rno-miR-19a, rno-miR-137, rno-miR-19b-2 and rno-miR-214) that exhibited at least a 2-fold change in the mean expression level following propofol treatment at all four time-points. In recent years, with the development of the next generation sequencing and chip array techniques combined with advances in bioinformatics, a great deal of data has been accumulated in relation to various physiological or pathological conditions. The potential value of these data has yet to be exploited fully. Our study provides a new method for re-use and re-analysis of these data for more effective and efficient application in different areas of research. In particular, publication of these data provides invaluable information for research groups with limited financial resources.

The results of the present study indicate that propofol may have the ability to regulate the expression of rno-miR-19a, rno-miR-137, rno-miR-19b-2 and rno-miR-214 and their target genes, ARC and EGR2. Additionally, this effect may last for at least 7 days after propofol exposure. However, the specific mechanism of these effects require further investigation, with particular reference the entire process of NSC development.

## Conclusion

The expression of four miRNAs (rno-miR-19a, rno-miR-137, rno-miR-19b-2 and rno-miR-214) and their target genes (EGR2 and ARC) were shown to be regulated by propofol in primary cultured embryonic NSCs. The underlying mechanism requires elucidation in more sophisticated studies.

## Additional files

Additional file 1: Table S3. Primers sequence of mRNAs and microRNAs. (XLSX 11 kb)
Additional file 2: Table S1. Gene list based on different search strategies. A total of 420 different genes that related to propofol exposure were selected when duplicates were excluded and their expression data were downloaded from the GEO Profiles. (XLSX 27 kb)
Additional file 3: Table S2. GO_BP of the different expressive genes. The genes were predicted to be involved in 124 biological functions. (XLSX 34 kb)
Additional file 4: Table S4. Predicted miRs of validated genes. By searching the four databases (miRWalk, miRanda, miRDB and TargetScan), 248 and 346 miRNAs were predicted to target the 3′ UTRs of EGR2 and ARC, respectively. (XLSX 42 kb)

## Abbreviations

ARC: Activity-regulated cytoskeleton-associated protein
bFGF: Fibroblast growth factor 2
BLAST: Basic local alignment search tool
BP: Biological process
cAMP: Cyclic adenosine monophosphate
CREB: cAMP responsive element binding protein
DAVID: The Database for Annotation Visualization and Integrated Discovery
DMSO: Dimethyl sulfoxide
EGF: Epidermal growth factor
EGR2: Early growth response-2
Ezh2: Enhancer of zeste 2 polycomb repressive complex 2 subunit
FoxO: Forkhead box sub-group O
FoxO1: Forkhead box O1
GEO: Gene expression omnibus
GO: Gene ontology
ICU: Intensive care unit
IGF: Insulin-like growth factor
LSD1: Lysine-specific histone demethylase lysine-specific demethylase 1
miRISC: miRNA-loaded RISC
NLRP3: NLR family pyrin domain containing 3
NSCs: The neural stem cells
PTEN: Phosphatase and tensin homolog
qRT-PCR: Quantitative real-time polymerase chain reaction
RISC: The RNA-induced silencing complex
RNA: Ribonucleic acid
SD: Sprague Dawley
STAT3: Signal transducer and activator of transcription 3
TLX: Nuclear receptor subfamily 2 group E
UTR: Untranslated rgion
